# Minimally Invasive Surgery: A Systematic Review of Laparoscopic and Robotic Techniques Across Common Surgical Procedures

**DOI:** 10.7759/cureus.110162

**Published:** 2026-06-03

**Authors:** Menpara Krishna Kishorbhai, Jegan Mohan S, Karthikhaeyan TR, Srishti Mohapatra, Roshan Chanchlani, Soham V Shah

**Affiliations:** 1 Department of Emergency Medicine, Padma Kuwarba Government Hospital, Rajkot, IND; 2 Department of General Surgery, Kasturba Medical College, Manipal Academy of Higher Education, Manipal, IND; 3 Department of General and Laparoscopic Surgery, Kovai Medical Center and Hospital (KMCH) Institute of Health Sciences and Research, Coimbatore, IND; 4 Department of Longevity and Regenerative Medicine, The Wellness Co., Gurgaon, IND; 5 Department of Pediatric Surgery, All India Institute of Medical Sciences Bhopal, Bhopal, IND; 6 Department of General Surgery, Dr. N. D. Desai Faculty of Medical Science and Research, Dharmsinh Desai University, Nadiad, IND

**Keywords:** laparoscopic surgery, minimally invasive surgery, oncologic outcomes, robotic surgery, surgical outcomes

## Abstract

Minimally invasive surgery has transformed surgical practice by reducing operative trauma, postoperative pain, and recovery time compared with open approaches. While conventional laparoscopy remains widely used across specialties, technical limitations such as restricted instrument articulation and limited visualization have driven the adoption of robotic-assisted surgery. This review aimed to synthesise contemporary comparative evidence evaluating robotic versus laparoscopic surgery, with emphasis on perioperative, functional, and oncologic outcomes. A systematic literature search identified comparative studies published between 2015 and 2025 involving adult patients undergoing robotic or laparoscopic procedures. Following screening and eligibility assessment, 11 studies were included in the qualitative synthesis. Data extraction focused on operative time, blood loss, conversion rates, complications, recovery parameters, functional outcomes, and oncologic adequacy. Owing to methodological heterogeneity, findings were synthesized descriptively and organized by outcome domain. Robotic surgery demonstrated selective advantages, including reduced conversion to open surgery, lower blood loss in specific procedures, and improved functional recovery in anatomically complex operations. Operative time was frequently longer with robotic techniques, while complication rates, hospital stay, and oncologic outcomes were generally comparable between approaches. The robotic surgery offers targeted benefits in complex, minimally invasive procedures while maintaining oncologic safety comparable to laparoscopy, supporting its role as a complementary surgical modality.

## Introduction and background

Minimally invasive surgery has revolutionized contemporary surgical practice by decreasing operative trauma, postoperative discomfort, and recovery duration compared with traditional open procedures [1]. Conventional laparoscopy is widely used across gynecology, general surgery, urology, and gastrointestinal oncology [2]. Despite its clinical value, laparoscopy has technical limitations, including restricted instrument articulation, two-dimensional visualization, reduced depth perception, and ergonomic challenges, which may affect precision in anatomically confined or technically demanding procedures [3,4].

The development of robotic-assisted surgery has been an attempt at overcoming some of these limitations, and robotic-assisted surgery is now increasingly being incorporated into minimally invasive surgical techniques [5]. The advantages offered by robots include articulated instrumentation, three-dimensional visualization, tremor filtering, dexterity, and ergonomic improvements for surgeons [6,7]. The integration of robotics into surgery has facilitated more complex abdominal and pelvic surgery, oncologic resections, and delicate dissections [8,9]. Some benefits of robotic surgery have included decreased intraoperative blood loss, fewer conversions to open surgery, and enhanced feasibility of selected complex surgeries [10-12]. On the other hand, there have also been documented increased operative times for robotic surgery, with similar results being achieved with laparoscopy in comparison studies [13,14].

Current literature consists of randomized controlled trials, observational comparisons, and systematic reviews by procedure type for robotic and laparoscopic surgery in various surgical specialties [12,14]. Interpretation is difficult due to variations in methodology, selection criteria, type of surgery, surgeon skill level, institution volume, learning curve effect, and outcomes definition. Most existing reviews have been confined to individual surgical specialties or procedures, thereby limiting their application to other surgical disciplines. Moreover, older systematic reviews may not be relevant to current practice due to advancements in robotic surgery and surgical experience gained since then.

The primary study question relates to the absence of a current and comprehensive cross-disciplinary synthesis that would be able to clearly delineate the clinical situations where robotic surgery offers a clinically relevant benefit compared to those where there is no difference in the results of both techniques. The purpose of this systematic review was to conduct a comprehensive synthesis of recent comparative literature regarding robotic versus laparoscopic surgery in different types of surgeries. The review considered the following perioperative, oncological, and functional outcomes: operation time, blood loss, length of hospital stay, complications, conversion, readmission, and reoperation; cancer-specific outcomes, such as tumor margin, lymph node harvest, recurrence, disease-free, and overall survival; and functional or recovery-related outcomes, including urinary function, sexual function, bowel recovery, renal functional preservation, and recovery-related outcomes.

Objectives of the review

The objective of this systematic review is to synthesize contemporary comparative evidence evaluating robotic and laparoscopic surgery across surgical specialties, with emphasis on perioperative, oncologic, and functional outcomes. Specifically, perioperative outcomes included operative time, intraoperative blood loss, length of hospital stay, postoperative complications, conversion to open surgery, readmission, and reoperation; oncologic outcomes included margin status, lymph node yield, recurrence, disease-free survival, and overall survival; and functional outcomes included urinary function, sexual function, bowel recovery, renal functional preservation, and postoperative recovery parameters. The review aims to delineate domains of advantage, equivalence, and limitation associated with robotic surgery to inform evidence-based clinical practice.

## Review

Methodology

Search Strategy

A systematic literature search was conducted in PubMed/Medical Literature Analysis and Retrieval System Online (MEDLINE), Scopus, Web of Science, Cochrane Library, and Google Scholar. For PubMed/MEDLINE, the following Boolean search string was used: (“robotic surgery” OR “robot-assisted surgery” OR “robotic-assisted surgery”) AND (“laparoscopy” OR “laparoscopic surgery” OR “conventional laparoscopy”) AND (“minimally invasive surgery” OR “operative time” OR “blood loss” OR “conversion” OR “complications” OR “hospital stay” OR “functional outcomes” OR “oncologic outcomes”). The search strategy was adapted for Scopus, Web of Science, Cochrane Library, and Google Scholar according to the search functions available in each database. Search limits included adult human studies, English-language publications, and articles published between 2015 and 2025. Reference lists of eligible studies and relevant articles were also manually screened to identify additional studies.

Eligibility Criteria

Studies were included if they involved adult human patients, were published in English between 2015 and 2025, directly compared robotic and laparoscopic surgical procedures, and reported at least one relevant outcome. Eligible perioperative outcomes included operative time, intraoperative blood loss, length of hospital stay, postoperative complications, conversion to open surgery, readmission, and reoperation. Eligible functional and recovery-related outcomes included urinary function, sexual function, bowel recovery, renal functional preservation, postoperative recovery parameters, and patient-reported recovery measures. Eligible oncologic outcomes included margin status, lymph node yield, recurrence, disease-free survival, and overall survival. Eligible study designs included randomized controlled trials and comparative observational studies, including prospective cohort studies, retrospective cohort studies, and propensity score-matched comparative studies. Studies involving benign or malignant surgical conditions were considered. Studies were excluded if they were non-comparative articles, reviews, editorials, case reports, case-control studies, prevalence studies, non-English publications, studies involving non-human subjects, or studies without sufficient relevant outcome data. Studies comparing minimally invasive surgery with open surgery alone were excluded unless they provided direct head-to-head data comparing robotic and laparoscopic approaches.

Study Selection

Titles and abstracts were independently screened by two reviewers, followed by full-text assessment against the predefined eligibility criteria. Disagreements during study selection were resolved through discussion and consensus. Study-selection results, including the number of records identified, screened, excluded, and included.

Data Extraction and Analysis

Data extraction and analysis were conducted in accordance with Preferred Reporting Items for Systematic Reviews and Meta-Analyses (PRISMA) 2020 guidelines [15]. Data were extracted using a standardized approach and included publication year, country or setting, study design, sample size, patient population, surgical procedure, comparator groups, outcomes assessed, and key comparative findings. Outcomes of interest included operative time, intraoperative blood loss, length of hospital stay, postoperative complications, conversion, readmission, reoperation, recovery parameters, functional outcomes, and oncologic outcomes where applicable. Because of heterogeneity in surgical specialty, procedure type, study design, patient population, follow-up duration, outcome definitions, and reported effect measures, quantitative meta-analysis, meta-regression, and subgroup meta-analysis were not performed. A subgroup meta-analysis comparing randomized trials with observational studies was not attempted because only two randomized trials were included, and they evaluated different procedures and outcome domains. Findings were therefore synthesized descriptively and presented in structured tables. For oncologic outcomes, equivalence and non-equivalence were interpreted descriptively rather than through formal equivalence testing. Equivalence indicated no statistically significant difference between robotic and laparoscopic approaches, or non-inferiority, as reported by the original study authors. References outside the included study set were used only for background context; the qualitative synthesis was based exclusively on the selected comparative studies.

Quality Assessment

The methodological quality of the included studies was assessed using a domain-based approach rather than a numerical scoring system. Assessment domains included clarity of study objectives, appropriateness of study design, comparability of robotic and laparoscopic groups, adequacy and objectivity of outcome measurement, completeness of follow-up, and control of potential confounding. Randomized trials were assessed with attention to randomization, allocation concealment, completeness of outcome reporting, and follow-up adequacy. Observational studies were assessed with attention to baseline group comparability, patient selection, adjustment methods such as propensity score matching, outcome reporting, and follow-up adequacy. These quality domains were incorporated into the study-level risk of bias judgement. No study was excluded solely based on methodological quality, and the quality assessment was used only to guide interpretation of the strength and limitations of the evidence.

Risk of Bias Assessment

Risk of bias was assessed at the study level using established domain-based criteria adapted from the Cochrane Risk of Bias tool (RoB 2) [16] for randomized controlled trials and the Risk of Bias In Non-randomized Studies - of Interventions (ROBINS-I) tool [17] for observational studies. The assessment was conducted independently by two assessors and focused on key domains, including selection bias, performance bias, detection bias, and reporting bias. In observational studies, particular attention was given to baseline comparability between robotic and laparoscopic groups, as well as the use of statistical methods such as propensity score matching or other adjustment techniques to minimize confounding. Randomized studies were assessed for adequacy of randomization, allocation concealment, blinding where applicable, and completeness of outcome reporting. Each assessor assigned an overall judgment of low, moderate, or high risk of bias for each included study. Disagreements were resolved through discussion and consensus.

Results

Search Results

**Figure 1:**
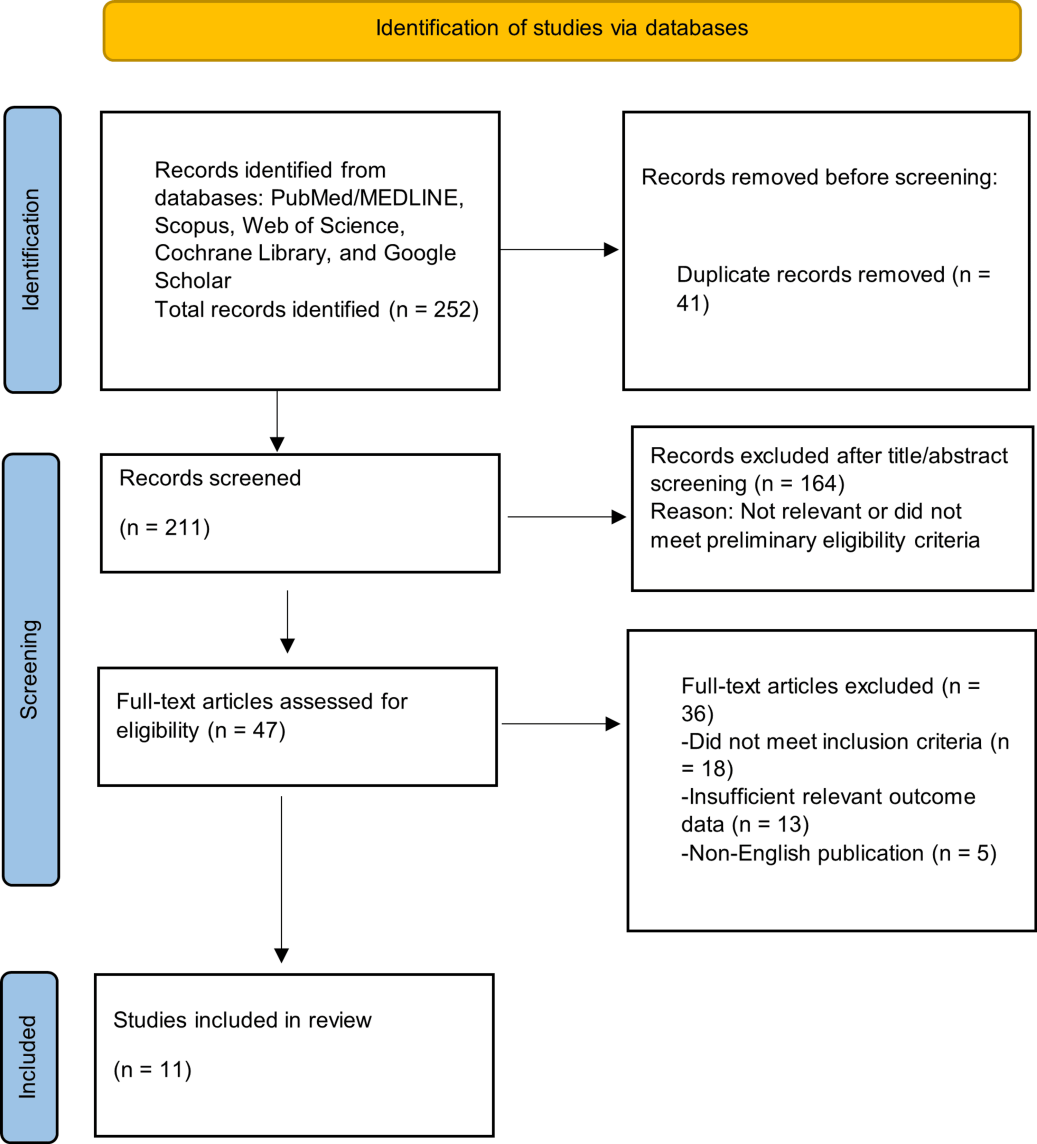
PRISMA flow diagram of the study selection PRISMA 2020 flow diagram depicting study identification, screening, eligibility, and inclusion. Records were retrieved from databases (n = 252), duplicates removed (n = 41), and studies screened and excluded based on predefined inclusion/exclusion criteria with documented reasons. Final studies (n = 11) were included for qualitative synthesis without meta-analysis due to heterogeneity. PRISMA: Preferred Reporting Items for Systematic Reviews and Meta-Analyses; MEDLINE: Medical Literature Analysis and Retrieval System Online

The database search identified 252 records. After the removal of 41 duplicate records, 211 records were screened by title and abstract. Of these, 164 records were excluded because they were not relevant to the review question or did not meet the preliminary eligibility criteria. A total of 47 full-text articles were assessed for eligibility. Of these, 36 full-text articles were excluded because they did not meet the inclusion criteria, had insufficient relevant outcome data, or were not published in English. Finally, 11 studies met all eligibility criteria and were included in the qualitative synthesis. The study selection process is summarized in the PRISMA flow diagram (Figure 1).

Study Characteristics

The review included 11 comparative studies evaluating robotic and laparoscopic surgical approaches across four surgical categories: gastrointestinal oncology, including right hemicolectomy, distal gastrectomy, and rectal cancer surgery, accounted for four studies (4/11, 36.4%); gynecologic surgery, including myomectomy and hysterectomy, accounted for three studies (3/11, 27.3%); general surgery, including ventral hernia repair and cholecystectomy, accounted for three studies (3/11, 27.3%); and urologic surgery, represented by partial nephrectomy, accounted for one study (1/11, 9.1%). The included studies addressed a mix of benign and malignant surgical indications. Benign conditions included myomectomy, benign hysterectomy, ventral hernia repair, and acute cholecystitis, whereas malignant or oncologic conditions included gastric cancer, rectal cancer, right hemicolectomy with complete mesocolic excision, and renal tumors. Reported outcomes included operative time, intraoperative blood loss, length of hospital stay, postoperative complications, conversion to open surgery, readmission, reoperation, renal functional preservation, urinary and sexual function, margin status, lymph node yield, recurrence, disease-free survival, and overall survival. Most included studies were observational comparative studies (9/11, 81.8%), while two studies were randomized trials (2/11, 18.2%). Comparative cohort designs accounted for six studies (6/11, 54.5%), including prospective and retrospective cohort studies. Propensity score matching was used in two studies (2/11, 18.2%) to improve baseline comparability between robotic and laparoscopic groups. These features of the included studies are summarized in Table 1.

**Table 1:** Characteristics and key comparative findings of included studies LM: laparoscopic myomectomy; RM: robotic myomectomy; LH: laparoscopic hysterectomy; RH: robotic hysterectomy; CLH: conventional laparoscopic hysterectomy; RC: robotic cholecystectomy; LC: laparoscopic cholecystectomy; LPN: laparoscopic partial nephrectomy; RAPN: robot-assisted partial nephrectomy; RDG: robotic distal gastrectomy; LDG: laparoscopic distal gastrectomy; RAP: robot-assisted proctectomy; 3D LAP: three-dimensional laparoscopic-assisted proctectomy; RR: robotic resection; LR: laparoscopic resection; OR: open resection; DFS: disease-free survival; OS: overall survival; WIT: warm ischemia time; EBL: estimated blood loss; TME: total mesorectal excision; NR: not reported

Study	Year	Country/setting	Surgical procedure/indication	Study design and sample size	Patient characteristics	Main outcomes assessed	Reported effect measure or key comparative result	Key findings
Kiran et al. [18]	2023	India; gynecology department of a multidisciplinary robotic surgery institute, Bengaluru/South India	Myomectomy for uterine fibroids	Retrospective observational study; LM n = 14, RM n = 21; total n = 35	Female patients undergoing myomectomy; mean age 33.7 ± 5.5 years in LM and 32.3 ± 5.6 years in RM	Operative time, estimated blood loss, hospital stay, hemoglobin drop, transfusion, conversion	Operative time: 184.6 ± 9.1 vs 300 ± 14.1 min; estimated blood loss: 395.7 ± 78 vs 255 ± 123.5 mL, p < 0.001; hospital stay: 3.9 ± 1.1 vs 2.2 ± 1.4 days, p < 0.001; conversion: one LM case converted to laparotomy, none in RM	Robotic myomectomy was associated with lower blood loss, shorter hospital stay, and no conversion to laparotomy, but longer operative time.
Takmaz and Güngör [19]	2020	Turkey; Acıbadem Mehmet Ali Aydınlar University Faculty of Medicine, İstanbul	Hysterectomy for benign gynecologic disease	Retrospective cohort study; LH n = 84, RH n = 62; total n = 146	Female patients undergoing hysterectomy for benign disease; mean age 51 ± 8.2 years in LH and 50 ± 4.5 years in RH	Operative time, estimated blood loss, hospital stay, first gas discharge, perioperative complications	Operative time: 105 ± 18 vs 150 ± 180 min, p < 0.01; EBL: 91 ± 65 vs 80 ± 37 mL, p = 0.43; hospital stay: 1.4 ± 0.5 vs 1.5 ± 0.7 days, p = 0.64	Robotic hysterectomy had a longer operative time, while blood loss, bowel recovery, and hospital stay were comparable.
Dhanani et al. [20]	2023	USA; multicenter trial at Lyndon B. Johnson General Hospital and Memorial Hermann Hospital System, Houston, Texas	Ventral hernia repair	Prospective multicenter blinded randomized controlled trial; robotic n = 65, laparoscopic n = 59; total randomized n = 124; two-year follow-up n = 101	Adult patients undergoing elective minimally invasive ventral hernia repair; most patients were female, Hispanic, and obese	Surgical site infection, surgical site occurrence, hernia recurrence, readmission, reoperation, mortality, patient-reported outcomes	Hernia recurrence: 4% robotic vs 13% laparoscopic, RR 0.3, 95% CI 0.06–1.39, p = 0.12; reoperation: 0% robotic vs 11% laparoscopic, p = 0.019	Robotic repair showed similar surgical site outcomes, with fewer reoperations and numerically lower recurrence at two years; findings were hypothesis-generating.
Tian et al. [21]	2023	China; multicenter study across three Chinese surgical departments	Right hemicolectomy with complete mesocolic excision for right-sided colon cancer	Retrospective multicenter propensity score-matched study; initial cohort n = 382; robotic n = 149, laparoscopic n = 233; after matching n = 142 per group	Adult patients with right-sided colon cancer; after matching, male/female distribution was 74/68 in robotic and 79/63 in laparoscopic groups; mean age 63.2 vs 63.4 years	Conversion, operative time, blood loss, bowel recovery, length of stay, complications, lymph node yield, DFS, OS, cost	Conversion: 0% vs 4.2%, p = 0.03; operative time: 200.9 vs 182.3 min, p < 0.001; lymph nodes: 20.4 vs 20.5, p = 0.861; two-year DFS: 84.9% vs 87.1%; OS: 83.8% vs 80.7%	Robotic surgery reduced conversion to open surgery but required longer operative time and higher cost; perioperative, pathologic, and oncologic outcomes were comparable.
Klein et al. [22]	2024	USA; level 1 trauma and tertiary referral medical centre, Westchester Medical Centre, New York	Cholecystectomy for acute cholecystitis	Retrospective cohort study; RC n = 130, LC n = 130; total n = 260	Adults aged ≥18 years; mean age 47 ± 18.3 years; 69.2% female	Operative time, conversion to open surgery, complications, readmission, mortality, and cholecystitis severity grading	Overall operative time similar: 105.82 ± 34.78 vs 108.12 ± 37.96 min, p = 0.612; conversion: 0.8% vs 1.5%, p = 0.19; shorter RC operative time in WJES grade B and C cases	Robotic cholecystectomy was comparable overall and showed shorter operative time in moderate and severe cholecystitis subgroups.
Marthandam et al. [23]	2024	India; Manipal Hospitals, Vijayawada	Cholecystectomy for acute cholecystitis by Parkland grade	Ambispective observational case-control study; RC n = 100, LC n = 100; total n = 200	Adults aged ≥18 years with acute cholecystitis; age reported by Parkland grade rather than surgical group	Operative time, intraoperative complications, conversion, postoperative complications, length of stay, readmission, cost, patient satisfaction	Intraoperative complications: 7 RC vs 17 LC, p = 0.01; conversion rates: 2 RC vs 7 LC, p = 0.01; bleeding: 7 RC vs 14 LC, p = 0.01; readmission: 2 RC vs 5 LC, p = 0.11	Robotic cholecystectomy showed fewer intraoperative complications and lower conversion rates, particularly in higher-grade cholecystitis, but with higher cost and longer setup/docking time.
Chen et al. [24]	2025	China; First Affiliated Hospital of Nanchang University	Partial nephrectomy for intermediate/high-complexity endophytic renal tumours	Propensity score-matched retrospective study; initial cohort n = 191; LPN n = 120, RAPN n = 71; after matching n = 70 per group	Adult patients with R.E.N.A.L. nephrometry score ≥7 endophytic renal tumours; after matching, mean age 48.7 vs 46.4 years; male sex 50.0% vs 51.4%	Operative time, warm ischemia time, blood loss, complications, renal function, trifecta/pentafecta, oncologic outcomes	WIT: 27.5 vs 23.5 min, p < 0.001; 48-hour eGFR decline: 21.0 vs 14.9 mL/min/1.73 m², p = 0.011; trifecta: 30% vs 60%, p < 0.001; complications not significantly different	Robot-assisted partial nephrectomy improved warm ischemia time, early renal functional preservation, and trifecta achievement, with comparable long-term renal and oncologic outcomes.
Lu et al. [25]	2024	China; Fujian Medical University Union Hospital	Distal gastrectomy for resectable gastric cancer	Randomized phase II trial; 300 randomized; modified intention-to-treat analysis RDG n = 141, LDG n = 142	Adult patients with resectable gastric cancer; mean age 59.4 vs 59.3 years; male sex 66.7% vs 63.4%	Three-year DFS, OS, recurrence, lymph node outcomes, postoperative recovery, complications, cost	Three-year DFS: 85.8% vs 73.2%, p = 0.011; HR for DFS 0.541, 95% CI 0.314–0.932; recurrence: 12.1% vs 21.1%, HR 0.546, 95% CI 0.302–0.990	Robotic distal gastrectomy met non-inferiority for three-year DFS and showed lower recurrence, with oncologic outcomes at least comparable to laparoscopy.
Liu et al. [26]	2025	China; Second Hospital of Dalian Medical University, Dalian	Robotic versus 3D laparoscopic resection for middle and low rectal cancer	Single-center retrospective cohort study; RAP n = 125, 3D LAP n = 110; total n = 235	Adult patients with middle and low rectal cancer; mean age 63.0 vs 64.0 years; male sex 63.2% vs 69.1%	Operative time, blood loss, cost, complications, lymph node retrieval, urinary function, sexual function, OS, DFS	Operative time: 162.0 ± 44.0 vs 149.0 ± 41.0 min, p = 0.034; blood loss: 51.0 ± 34.0 vs 63.0 ± 43.5 mL, p = 0.010; urinary recovery OR 3.45, 95% CI 1.82–6.54, p < 0.001; male sexual recovery OR 2.89, p = 0.004; female sexual recovery OR 3.12, p = 0.017	Robotic rectal surgery had longer operative time and higher cost but lower blood loss and better urinary and sexual functional recovery, with comparable survival outcomes.
Madarasz et al. [27]	2025	Germany, University Hospital OWL Campus Lippe, Bielefeld University	Rectal cancer surgery: robotic, laparoscopic, and open resection	Single-center retrospective comparative study; RR n = 62, LR n = 68, OR n = 82; total n = 212	Adult patients with histologically confirmed rectal cancer; mean age 68.1 years in RR, 65.1 in LR, 69.3 in OR; male sex 72.6% in RR, 60.3% in LR, 62.2% in OR	TME quality, operative time, hospital stay, conversion, complications, lymph node yield, OS, DFS	Conversion: RR 3.2% vs LR 14.7%; hospital stay: RR 10 days vs LR/OR 14 days; operative time: RR 304 min vs LR 221 min and OR 222 min; five-year OS and DFS did not differ notably	Robotic surgery showed lower conversion and shorter hospitalisation, with longer operative time and comparable oncologic survival.
Jeong et al. [28]	2022	Republic of Korea; Kangnam Sacred-Heart Hospital, Hallym University Medical Centre, Seoul	Hysterectomy for a large uterus with benign gynecologic disease	Retrospective cohort study; RH n = 197, CLH n = 200; total n = 397	Female patients with benign gynecologic disease and uterine weight >250 g; median age 47 years in both groups	Hospital stay, EBL, operative time, conversion, intraoperative and postoperative complications	Hospital stay: 5 days in both groups; EBL: 100 vs 150 mL; operative time: 120 min in both groups; conversion: 0% vs 0.5%, p = 0.320; complication rates not significantly different	Robotic hysterectomy was not inferior to conventional laparoscopic hysterectomy for large benign uteri, with comparable perioperative and immediate postoperative outcomes.

Risk of Bias Assessment

The risk of bias of the included studies was assessed to identify potential sources of systematic error. As most studies were retrospective or observational, selection bias related to non-randomized allocation and baseline differences was a primary concern. Performance bias was considered due to variability in surgeon experience and learning curves, while detection bias was generally low given the objective reporting of outcomes. Studies using propensity score matching showed reduced selection bias, and the randomized controlled trials demonstrated low overall risk of bias. Most studies were judged to have a moderate overall risk of bias, which was considered when interpreting the findings. The independent assessors' assessments and final consensus judgments are presented in Table 2.

**Table 2:** Risk of bias assessment of included studies Risk of bias assessment of included studies across selection, performance, and detection domains. Two assessors independently applied criteria adapted from RoB 2 and ROBINS-I, with disagreements resolved by consensus. The table presents independent assessments and final consensus judgments. ROBINS-I: Risk of Bias In Non-randomized Studies – of Interventions

Study	Study design	Selection bias	Performance bias	Detection bias	Assessor 1 overall assessment	Assessor 2 overall assessment	Final consensus risk of bias
Kiran et al. [18]	Retrospective observational	Moderate	Moderate	Low	Moderate	Moderate	Moderate
Takmaz and Güngör [19]	Retrospective cohort	Moderate	Moderate	Low	Moderate	Moderate	Moderate
Dhanani et al. [20]	Multicenter blinded randomized controlled trial	Low	Moderate	Low	Low	Low	Low
Tian et al. [21]	Multicenter retrospective propensity-matched	Low-Moderate	Moderate	Low	Moderate	Moderate	Moderate
Klein et al. [22]	Retrospective cohort	Moderate	Moderate	Low	Moderate	Moderate	Moderate
Marthandam et al. [23]	Observational ambispective	Moderate	Moderate	Low	Moderate	Moderate	Moderate
Chen et al. [24]	Propensity score-matched retrospective	Low-Moderate	Moderate	Low	Moderate	Moderate	Moderate
Lu et al. [25]	Randomized controlled trial	Low	Low	Low	Low	Low	Low
Liu et al. [26]	Single-centre retrospective comparative	Moderate	Moderate	Low	Moderate	Moderate	Moderate
Madarasz et al. [27]	Retrospective three-arm comparative	Moderate	Moderate	Low	Moderate	Moderate	Moderate
Jeong et al. [28]	Retrospective cohort	Moderate	Moderate	Low	Moderate	Moderate	Moderate

Perioperative Outcomes

Across the 11 included studies, perioperative and early recovery outcomes were reported using procedure-specific measures, including operative time, intraoperative blood loss, length of hospital stay, postoperative complications, conversion to open surgery, readmission, reoperation, surgical site outcomes, warm ischemia time, and early postoperative organ function where applicable. Robotic surgery showed selected advantages in specific procedures, including reduced blood loss, lower conversion rates, fewer intraoperative complications, shorter warm ischemia times, and shorter hospital stays in some studies. Operative time was longer in several robotic procedures, while complication rates were generally comparable between robotic and laparoscopic approaches. Table 3 summarizes the perioperative and early recovery findings reported across all 11 included studies.

**Table 3:** Perioperative and early recovery outcomes of robotic versus laparoscopic surgery Summary of perioperative and early recovery outcomes reported across all included comparative studies (n = 11), organized by surgical procedure. Outcomes included operative time, blood loss, length of hospital stay, postoperative complications, conversion to open surgery, readmission, reoperation, surgical site outcomes, warm ischemia time, and early postoperative recovery measures. No statistical pooling was performed because of heterogeneity in study design, surgical procedures, and outcome reporting.

Surgical procedure	Perioperative or early recovery outcomes assessed	Robotic surgery outcomes compared with laparoscopy	References
Myomectomy	Operative time, blood loss, length of hospital stay	Reduced blood loss and shorter hospital stay; longer operative time	[18]
Hysterectomy for benign disease	Operative time, blood loss, length of hospital stay, bowel recovery	Longer operative time, blood loss, length of stay, and postoperative recovery were comparable	[19]
Ventral hernia repair	Surgical site infection, surgical site occurrence, recurrence, readmission, reoperation, mortality, patient-reported outcomes	No significant differences in surgical site infection or surgical site occurrence; no reoperations occurred in the robotic group compared with the laparoscopic group	[20]
Right hemicolectomy with complete mesocolic excision	Conversion to open surgery and perioperative outcomes	Lower conversion rate to open surgery; perioperative outcomes were comparable	[21]
Acute cholecystitis	Operative time, conversion rate, complications	Comparable operative time and similar conversion and complication rates, with potential advantages in complex cases	[22]
Acute cholecystitis assessed by Parkland grading	Operative time, complications, length of stay, readmission	Fewer intraoperative complications and lower readmission rates, particularly in higher Parkland grades	[23]
Partial nephrectomy	Warm ischemia time, renal function, complications	Shorter warm ischemia time and better early postoperative renal function; complication rates were similar	[24]
Distal gastrectomy for gastric cancer	Postoperative complications	Comparable postoperative complication rates	[25]
Rectal cancer resection using robotic surgery versus 3D laparoscopy	Blood loss, complications, postoperative urinary and sexual function	Reduced intraoperative blood loss and improved postoperative urinary and sexual function; complications were similar	[26]
Rectal cancer surgery	Operative time, length of hospital stay, conversion	Lower conversion rates and shorter hospital stays	[27]
Hysterectomy for large uterus	Operative time, blood loss, perioperative complications	Comparable perioperative outcomes despite increased surgical complexity	[28]

**Figure 2:**
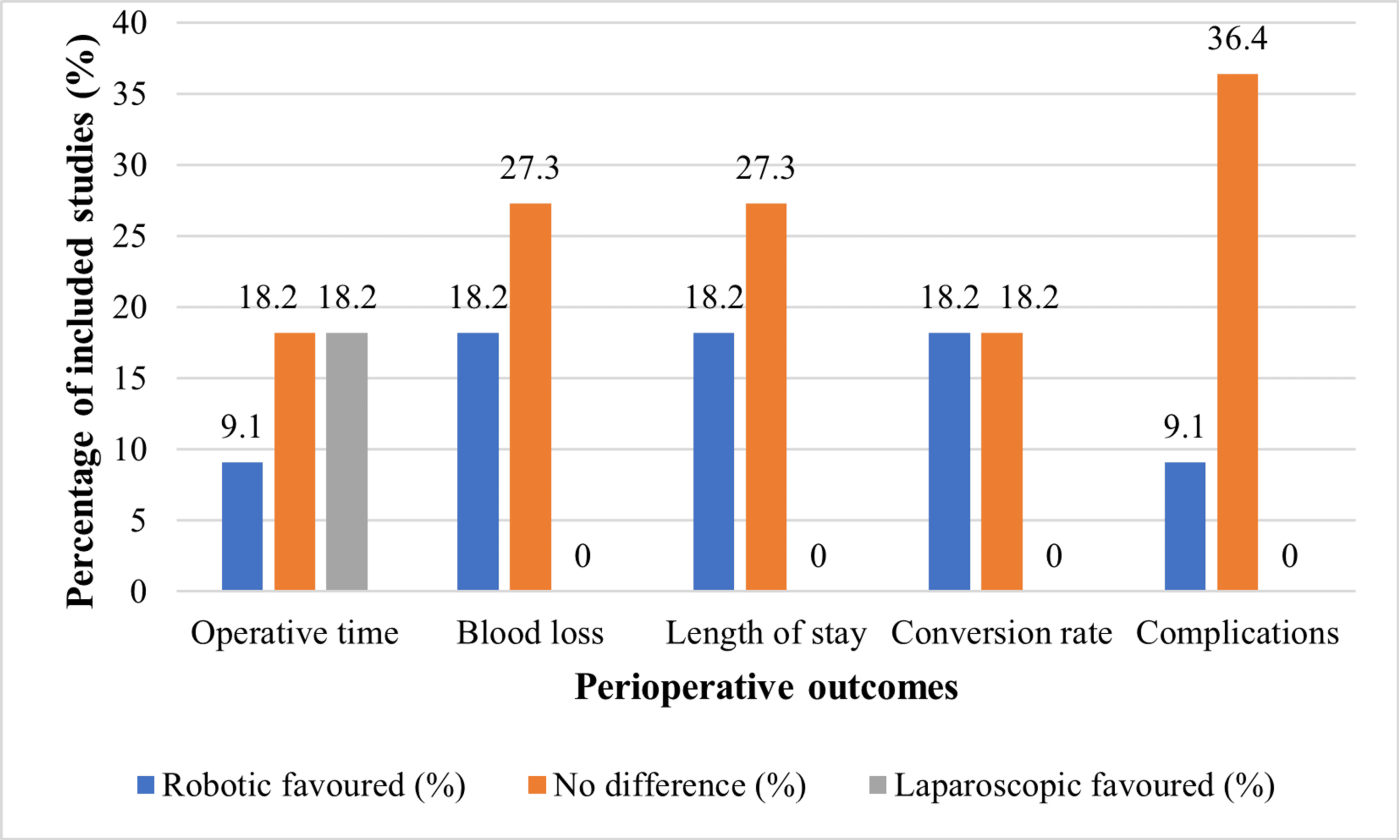
Comparative perioperative outcomes of robotic and laparoscopic surgery Bar chart showing the distribution of included studies (n = 11) reporting perioperative outcomes favouring robotic surgery, no difference, or favouring laparoscopy across key parameters. Percentages were calculated based on the number of studies reporting each outcome category for each parameter. Data were synthesized qualitatively without statistical pooling due to heterogeneity.

Figure 2 shows the distribution of perioperative outcomes across included studies, showing the percentage of studies favoring robotic surgery, reporting no significant difference, or favoring laparoscopy for each outcome.

Oncologic Outcomes

**Figure 3:**
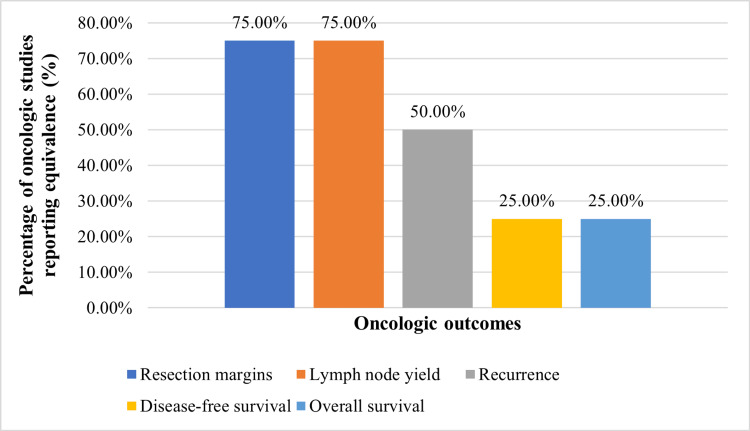
Oncologic outcome equivalence between robotic and laparoscopic surgery Bar chart showing the proportion of oncologic studies reporting equivalent oncologic outcomes between robotic and laparoscopic surgery. Percentages were calculated using studies with malignant or oncologic surgical indications as the denominator (n = 4), not the total number of included studies. Labels above bars show the number of studies and the corresponding percentage. Equivalence was defined descriptively as no reported statistically significant difference between robotic and laparoscopic approaches or as non-inferiority reported by the original study authors. No statistical pooling was performed because of heterogeneity in study design, procedures, and outcome reporting.

Among studies involving malignant or oncologic surgical indications, robotic and laparoscopic approaches demonstrated broadly comparable oncologic adequacy. Reported oncologic endpoints included resection margins, lymph node yield, recurrence, disease-free survival, and overall survival. Across gastric and colorectal cancer procedures, robotic surgery showed no clear oncologic disadvantage compared with laparoscopy. In some studies, lower conversion rates were reported with robotic surgery without evidence of compromised oncologic outcomes. To avoid mixing benign and oncologic indications, oncologic outcome proportions were calculated only among studies involving malignant or oncologic surgical indications. Figure 3 summarizes the proportion of oncologic studies reporting equivalent outcomes across the reported oncologic endpoints.

Functional and Recovery Outcomes

Other reports indicated better functional recovery after robotic surgery, especially in rectal cancer resections, where urinary and sexual functional outcomes were high. Parameters about recovery (bowel function) revert, and readmission was overall comparable, but robotic surgery showed lower readmission and complication rates in higher grades of disease in a few studies. All the measures of length of stay and postoperative recovery showed no significant differences between methods, which suggests that the advantages of robotic surgery in terms of functionality can be the most tangible in anatomically complicated operations. Table 4 shows the functional and postoperative recovery outcomes.

**Table 4:** Functional and recovery outcomes of robotic versus laparoscopic surgery Summary of functional and postoperative recovery outcomes across included studies, organized by surgical procedure and outcome measures. Data were extracted from comparative studies (n = 11) and synthesized qualitatively, including parameters such as organ-specific function, complications, and readmission. No statistical pooling was performed due to heterogeneity in study design and outcome reporting.

Surgical procedure	Functional or recovery outcomes assessed	Robotic surgery outcomes compared with laparoscopy	Reference
Rectal cancer resection	Urinary and sexual function	Improved postoperative urinary and sexual function	[26]
Acute cholecystitis	Complications, readmission	Fewer intraoperative complications and lower readmission	[19]
Partial nephrectomy	Renal function, ischemia time	Shorter warm ischemia time and better early renal function	[24]
Hysterectomy (large uterus)	Perioperative recovery, complications	Comparable perioperative outcomes despite increased complexity	[28]

Discussion

The review is based on 11 comparative studies that involved gynecology, general surgery, urology, and gastrointestinal oncology as evidence, and it offers a systematic assessment of robotic and laparoscopic interventions. The results show that robotic surgery provides procedure-specific perioperative and functional benefits and demonstrates oncologic outcomes comparable to laparoscopy across malignant indications. Outcome perioperative data show that there are intraoperative studies of uniform results. Robotic myomectomy was also linked with reduced intraoperative blood loss and reduced hospitalization, as indicated in Table 1, and was also linked with an increased operative period. The same tendencies were also noted in gynecologic hysterectomy and benign operating cases, where the time spent at the operating table was more active in the robotic groups, and blood loss and recovery indicators were similar. In the analyzed literature, a reduced conversion to open surgery was identified as a significant benefit of robotic surgery, specifically in technically challenging colorectal and hepatobiliary surgeries. This decrease in conversion rates in robotic right hemicolectomy and rectal cancer resections, as indicated by Table 3, is indicative of increased dexterity and visualization of anatomical planes confined to a standard. The clinical relevance of robotic platforms is further explained by functional and recovery outcomes. Table 4 in the summary indicated that robotic rectal cancer surgery had higher postoperative urinary and sexual functioning, and robotic partial nephrectomy had a short period of warm ischemia with better early kidney functioning.

These results indicate that articulations and refined methods of instruments and a stable three-dimensional visualization play a part in preserving key structures. There was little difference in the length of hospital stay and total complication rates among approaches to most procedures, suggesting that functional advantages are most apparent in anatomically complicated surgeries instead of routine cases. Robotic surgery in oncologic surgery was highly involved in gastric and colorectal surgery, showing non-inferiority. The measures of margin status, lymph node yield, recurrence, disease-free survival, and long-term survival did not show any significant difference between robotic and laparoscopic surgery. Notably, conversely, oncologic competence was reached, and a reduction of conversion rates in robotic groups was achieved, which supports the capability of robotic systems to preserve their oncologic competence in complicated resections.

The results indicate a selective and context-dependent place of robotic surgery in the present-day modern practice of minimally invasive surgery. Operations with small pelvic areas, severe inflammation, or high technical demand seem to be the most promising with robotic assistance [29,30]. Decreased conversion and better functional outcome in these environments raise the opportunity of downstream benefits of patient quality of life and postoperative recovery [31]. Comparatively, less technically intensive standard procedures show a similar result between the robotic and laparoscopic methods, which supports the further use of traditional laparoscopy. This aspect implies working room efficiency and resource utilization. These metrics are likely to be affected by the experience of the surgeon, institutional volume, and knowledge of robotic platforms [32]. The results show that the operative period needs to be discussed in conjunction with the measures of safety, functional maintenance, and technical feasibility as opposed to separately. The oncologic equivalence between robotic surgery and various forms of cancer makes it more acceptable that robotic surgery is a viable option in oncologic care with minimal invasiveness [33,34]. Proper maintenance of oncologic quality and low conversion rates justify the utilization of robotic platforms in the treatment of intricate malignancies, given that the necessary experience and patient criteria are considered.

The patterns of results found in this review can be connected with more extensive tendencies presented in the literature on minimally invasive surgery. Comparative studies on the different surgical specialties have always shown the similarity between perioperative morbidity and oncologic adequacy between robotic and laparoscopic methods [35]. Preferential benefits are given to robotic surgery in technically challenging situations, especially in the case of pelvic dissection and organ-sparing surgeries [36,37]. The repeated finding of increased operative time and the like complication rate is a balance between technical competence and operational efficiency [38]. The uneven distribution of reported benefits in procedures points to the role of case complexity and surgical situation, and not the inherent excellence of a given modality [39,40]. The systematic review offered in the current study, incorporating perioperative, functional, and oncologic spheres, gives a sense of what needs to be measured and what needs to remain the same across specialities.

The findings of this review highlight the importance of matching surgical approach selection to procedural complexity and clinical context. Robotic surgery does not demonstrate universal superiority but offers distinct advantages in technically demanding procedures that require precise dissection, enhanced visualization, and functional preservation [41]. These benefits are most apparent in anatomically confined spaces and complex oncologic resections. Perioperative safety outcomes are largely comparable between robotic and laparoscopic approaches, indicating that both techniques remain viable options in minimally invasive surgery. The clinical value of robotic platforms becomes most relevant when reduced conversion rates, improved functional outcomes, or technical feasibility offset longer operative times. Importantly, oncologic outcomes across malignant indications remain comparable, supporting the selective integration of robotic surgery into cancer care based on patient characteristics, surgeon expertise, and institutional experience.

Limitations and Future Directions

A key limitation of this review is its deliberately narrow focus on direct comparisons between robotic and conventional laparoscopic surgery. Landmark trials comparing minimally invasive surgery with open surgery were not included unless they specifically provided head-to-head robotic-versus-laparoscopic data. This restriction was applied to maintain methodological consistency, but it also reduced the number of eligible studies. Most included studies were retrospective or observational, which may increase selection bias and limit causal interpretation. Comparability was further affected by heterogeneity in surgical procedures, patient populations, outcome definitions, surgeon experience, institutional volume, and robotic learning curves. These differences limited the feasibility of quantitative pooling and required descriptive synthesis. Limited reporting of cost-effectiveness, long-term functional outcomes, and quality-of-life measures also restricted the overall assessment.

The future directions would focus more on expertly designed prospective and multicenter studies, especially in the case of procedures that are not well-proceded. Perioperative, functional, and quality-of-life outcomes should have standardized definitions to improve the level of consistency in studies. Long-term oncologic follow-up is required in order to demystify long-term disease control. Sustainable adoption will be informed by the incorporation of cost-effectiveness and resource utilization analyses. Evidence-based clinical implementation can also be enhanced by systematic assessments of learning curves and rising robotic technologies.

## Conclusions

This review indicates that robotic surgery provides outcomes comparable to conventional laparoscopy in perioperative and oncologic domains, with selective advantages in technically complex and anatomically confined procedures. Reduced conversion rates, lower blood loss in selected cases, and improved functional recovery highlight its value in specific clinical contexts, although longer operative time remains a limitation. Robotic surgery should therefore be considered a complementary approach rather than a universal replacement, with its optimal use guided by procedural complexity, surgeon expertise, and institutional resources. Future research should prioritize high-quality prospective studies with standardized outcomes and long-term follow-up to better define its role in minimally invasive surgical practice.
